# Relationship between hyperlipidemia and lumbar disc degeneration in patients with low back pain: an observational retrospective study

**DOI:** 10.3389/fphys.2026.1810691

**Published:** 2026-06-03

**Authors:** Shuanchi Wang, Linfeng Wang, Da Shi, Jiangtao Yan, Xiong Zhang, Rao Zhang

**Affiliations:** Hebei Medical University Third Hospital, Shijiazhuang, China

**Keywords:** hyperlipidemia, intervertebral disc, lipid metabolism, low back pain, lumbar disc degeneration

## Abstract

**Objective:**

To investigate the association between hyperlipidemia lumbar disc degeneration (LDD) in patients hospitalized for low back pain.

**Methods:**

A total of 165 patients with hyperlipidemia hospitalized for low back pain (mean age 44.06 ± 9.69 years; BMI 25.95 ± 2.55 kg/m²; 59.39% male) were enrolled and compared with 165 age-, sex-, and BMI-matched non-hyperlipidemic controls. The Pfirrmann grading system was utilized to evaluate the severity of LDD in both groups. The primary outcome was the difference in radiographic features of disc degeneration between groups, while secondary outcomes assessed the correlation between specific lipid profiles and the severity of LDD.

**Results:**

The hyperlipidemia group exhibited a significantly higher number of degenerated discs compared to the non-hyperlipidemia group (P<0.05), with significant differences observed particularly at the L1/2, L2/3, and L3/4 levels (P<0.05). The number of severely degenerated discs was also significantly higher in the hyperlipidemia group (P = 0.008), most notably at the L1/2 and L2/3 levels (P = 0.044 and P = 0.026, respectively). Furthermore, the prevalence of multi-level LDD was significantly higher in the hyperlipidemia group (P<0.05). While there was no significant difference in the number of patients with 3-level degeneration between the two groups (P = 0.238), significant differences were found in 4-level and 5-level degeneration (P<0.05). Abnormalities in Total Cholesterol (TC), Triglycerides (TG), and Lipoprotein(a) [Lp(a)] were associated with LDD (P<0.05); however, no significant difference in LDD severity was found among the four subtypes of hyperlipidemia (hypercholesterolemia, hypertriglyceridemia, mixed hyperlipidemia, and low HDL cholesterolemia) (P>0.05).

**Conclusion:**

This study demonstrates a significant association between hyperlipidemia and lumbar disc degeneration. Patients with hyperlipidemia hospitalized for low back pain present with a greater number of degenerated discs, a higher propensity for multi-level degeneration, and a significantly higher proportion of severe degeneration. Elevated levels of TC, TG, and Lp(a) are correlated with the severity of lumbar disc degeneration. Future research is needed to conduct a forward-looking study to determine whether lipid regulation has a preventive or therapeutic effect on intervertebral disc-related low back pain.

## Introduction

Low back pain (LBP) is defined as localized pain in the lumbar region lasting at least one day, with or without radiating pain to the lower extremities. With the global aging population, LBP has become a highly prevalent condition and a leading cause of disability worldwide. Within the UK National Health Service (NHS), back pain alone accounts for 40% of sickness absence, costing the British economy £10 billion annually (Kabeer et al., 2023). In the United States, the total direct medical costs associated with LBP reached $26.3 billion in 1998 (Urits et al., 2019). The etiology of LBP is multifactorial, encompassing muscle strain, fibrositis, LDD, and lumbar instability. Among these, LDD is recognized as a primary cause, accounting for approximately 26% to 42% of chronic LBP cases (Katz, 2006; Peng, 2013).

Anatomically, the intervertebral disc consists of the superior and inferior cartilaginous endplates, the annulus fibrosus (AF), and the nucleus pulposus (NP). It facilitates multi-directional spinal movement and performs critical biomechanical functions, such as maintaining spinal stability and absorbing external mechanical shocks (Wang et al., 2022; Koroth et al., 2023). Traditionally, LDD was attributed to mechanical factors, including compressive loading, shear stress, and vibration. However, recent studies have confirmed that LDD is a multifactorial pathological process. Various pathways, including microRNA dysregulation, lipid metabolism disorders, and iron deficiency-induced apoptosis, have been shown to trigger disc degeneration (Furukawa et al., 2004; Bing et al., 2024; Zhou et al., 2024; Chang et al., 2025; Liu et al., 2025). Furthermore, several risk factors for LDD have been identified; while the association with aging is well-established, other factors such as smoking, obesity, diabetes, hypertension, and osteoporosis may also contribute to the progression of lumbar disc degeneration (Liuke et al., 2005; Jang et al., 2016; Teraguchi et al., 2016; Xiao et al., 2018; Alpantaki et al., 2019; Kiraz and Demir, 2020).

Lipid metabolism encompasses the enzymatic processes of lipid digestion, absorption, synthesis, and catabolism. Perturbation of lipid homeostasis alters the disc microenvironment; in pathological states, dyslipidemia can trigger obesity-related disorders such as hyperlipidemia, diabetes, and atherosclerosis (Ipsen et al., 2016; Padró et al., 2018). Characterized by the release of detrimental adipokines and the infiltration of fatty acids and cholesterol in circulation and tissues, lipid metabolism disorders participate in the pathological process of disc degeneration by orchestrating the inflammatory microenvironment, enhancing catabolic activity, promoting cartilage calcification, and inducing apoptosis of nucleus pulposus (NP), annulus fibrosus (AF), and cartilaginous endplate cells (Higashino et al., 2007; Curic, 2020).

As the largest avascular structure in the human body, the intervertebral disc relies primarily on diffusion through the cartilaginous endplates for nutrition. Elevated total cholesterol (TC) and serum triglycerides (TG)—established risk factors for atherosclerosis—can further compromise the already limited blood supply, leading to nutritional insufficiency and ultimately triggering progressive structural degeneration of the disc (Kurunlahti et al., 1999; Kauppila, 2009). Conversely, elevating high-density lipoprotein (HDL) or lowering TG levels may alleviate pathological alterations associated with disc degeneration.

Emerging evidence indicates that oxidized low-density lipoprotein (ox-LDL) and its receptor LOX-1 promote the expression of matrix metalloproteinase 13 (MMP-13) by activating the NF-κB signaling pathway, thereby accelerating the degradation of the extracellular matrix (ECM) (Bing et al., 2024; Zhou et al., 2024). Furthermore, dyslipidemia influences the initiation and development of disc degeneration via oxidative stress. Specifically, ox-LDL promotes the overexpression of Dynamin-related protein 1 (Drp-1) and the generation of mitochondrial reactive oxygen species (ROS) in disc cells (Li et al., 2017; Lin et al., 2025; Liu et al., 2025; Wu et al., 2025). According to the free radical theory of aging, the functional decline of human tissues and organs is closely related to ROS-induced oxidative stress (Venkataraman et al., 2013). Adipose tissue exacerbates this stress, as lipid accumulation leads to excessive ROS production in adipocytes, and elevated free fatty acids maintain a state of chronic oxidative stress (Furukawa et al., 2004; Matsuda and Shimomura, 2013).

Lipocalin, produced by adipose tissue, is expressed at low levels in the intervertebral disc. Studies have shown a negative correlation between lipocalin expression in degenerated disc tissue and the Pfirrmann grade; specifically, lipocalin expression is downregulated in nucleus pulposus cells of degenerated discs, while its receptors, adiponectin receptor 1 (adipoR1) and receptor 2 (adipoR2), are upregulated. Lipocalin may exert a protective effect against lumbar disc degeneration by inhibiting inflammatory responses (Sharma, 2018; Zhang et al., 2025).

Despite these compelling findings suggesting the involvement of lipid metabolism in spinal degeneration, it remains unclear whether radiographic characteristics of degenerated discs differ between patients with and without hyperlipidemia. Therefore, the present study aims to evaluate the morphological characteristics of disc degeneration via magnetic resonance imaging (MRI) to elucidate the impact of hyperlipidemia on disc health.

## Materials and methods

### Study design and participants

This observational retrospective study was approved by the Institutional Ethics Committee of our hospital (Approval No.: KE 2022-022-1). All clinical and imaging data of eligible low back pain inpatients admitted between January 2021 and December 2024 were retrospectively analyzed based on existing medical records, without prospective active recruitment of additional patients.

Inclusion criteria were as follows: 1. Age ranging from 20 to 59 years. 2.Patients presenting with low back pain, with or without lower limb symptoms. 3.Patients whose complaints of low back pain, with or without associated radicular symptoms (such as radicular pain, numbness, and fatigue), persisted for more than 12 weeks despite conservative treatment. 4.Patients who completed both lumbar spinal magnetic resonance imaging (MRI) and serum lipid testing.

Exclusion criteria:1.History of spinal surgery 2. Spinal fracture, Spinal fractures and infections 3. Malignant tumors 4. Rheumatoid arthritis 5. Congenital spinal deformities 6. Lumbar spondylolisthesis 7. Idiopathic scoliosis 8. Patients with hyperlipidemia who were on long-term lipid-lowering medication.

### Laboratory measurements and group assignment

All participants underwent venous blood collection in the morning after an overnight fast. The following serum lipid parameters were extracted from the medical records: total cholesterol (TC), triglycerides (TG), high-density lipoprotein cholesterol (HDL-C), low-density lipoprotein cholesterol (LDL-C), apolipoprotein A1 (ApoA1), apolipoprotein B (ApoB), and lipoprotein(a) Lp(a).

Patients were assigned to the case group based on a confirmed diagnosis of hyperlipidemia derived from the lipid test results. The diagnostic criteria for hyperlipidemia strictly adhered to the Chinese Guidelines for the Management of Blood Lipids. A diagnosis was established if any of the following thresholds were met: TC ≥ 5.2 mmol/L; LDL-C ≥ 3.4 mmol/L; HDL-C < 1.0 mmol/L; TG ≥ 1.7 mmol/L; or Lp(a) ≥ 300 mg/L. Patients in the control group were selected from the pool of LBP patients without hyperlipidemia and were matched to the case group at a 1:1 ratio using simple nearest-neighbor individual matching (not propensity score matching), with matching variables including age, sex, and body mass index (BMI).

### Study population and flow

**Figure 1:**
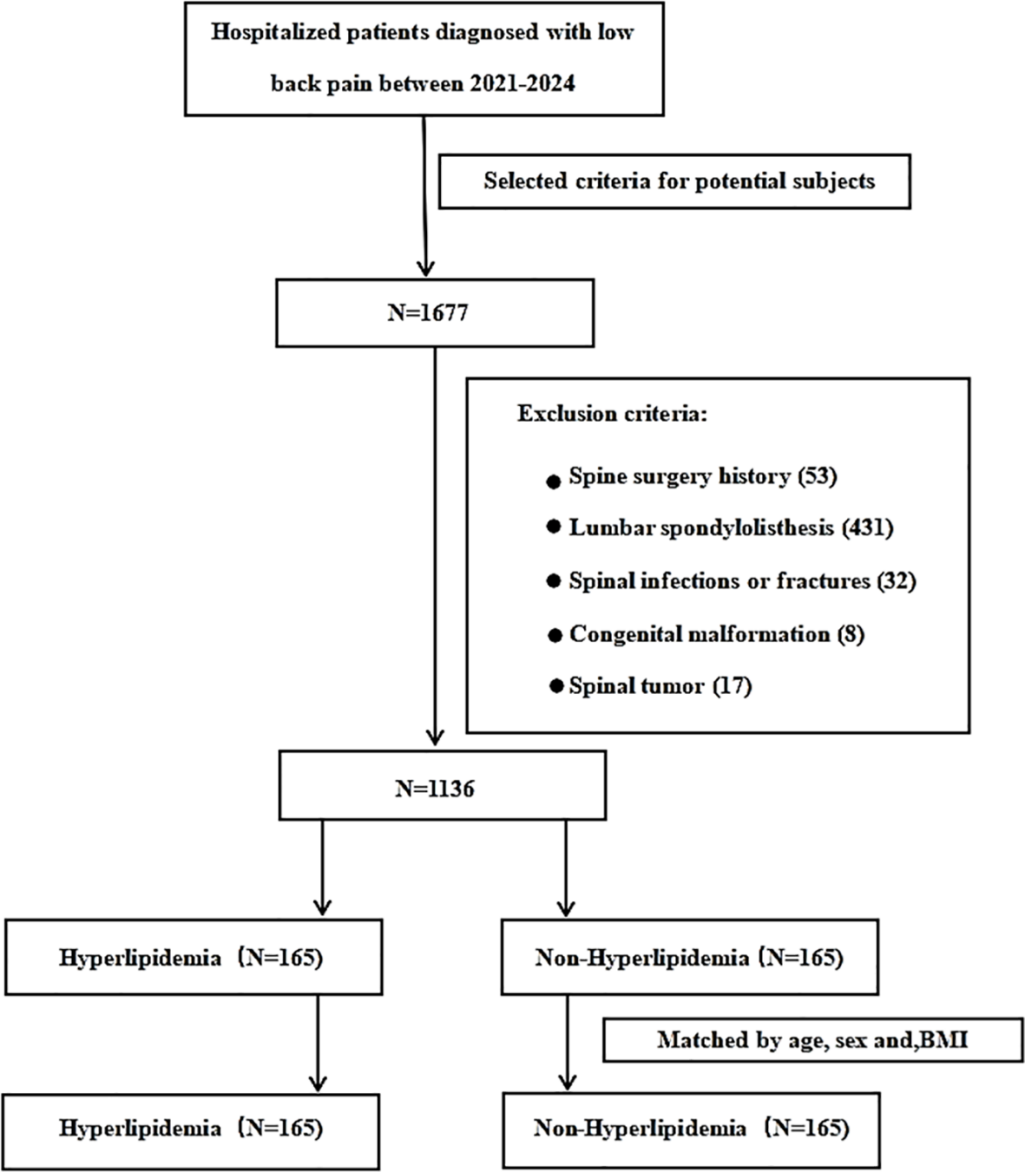
The flow chart of patient selection. The patient selection flowchart (**Figure 1**) shows the 1:1 simple nearest-neighbor individual matching process with age, sex, and BMI as matching variables, which is consistent with the detailed matching procedure described in the text.

The study initially included 2,108 hospitalized LBP patients admitted between 2021 and 2024. Of these, 431 patients were excluded due to incomplete imaging or laboratory data. Among the remaining 1,677 patients, a further 540 patients were excluded based on the exclusion criteria (53 with a history of spinal surgery, 431 with lumbar spondylolisthesis, 32 with spinal fractures/infections, 8 with congenital malformations, and 17 with tumors). Ultimately, 1,136 patients were included in the final analysis, of whom 165 were diagnosed with hyperlipidemia. The patient screening flowchart is presented in **Figure 1**.

To minimize selection bias and ensure baseline comparability, this study employed a 1:1 simple nearest-neighbor individual matching method, with matching variables including age, sex, and body mass index (BMI). Initially, all research subjects were rigorously screened according to the inclusion and exclusion criteria, and the study population was subsequently stratified into the hyperlipidemia group (case group) and the non-hyperlipidemia group (potential control group). The matching process was anchored on the case group: for each patient in the hyperlipidemia group, 1:1 nearest-neighbor matching was performed using the database of eligible non-hyperlipidemia inpatients. The matching criteria were strictly defined as follows: exact matching for sex; age tolerance range of ±2 years; BMI tolerance range of ±2 kg/m². Priority was given to selecting individuals with the smallest difference in baseline characteristics (age, sex, BMI) to serve as controls. The matching process strictly adhered to the one-to-one non-replacement principle: each control subject was matched to only one case and was not reused. This procedure successfully achieved balanced baseline characteristics for age, sex, and BMI between the two groups, and any remaining potential confounding factors were subjected to subsequent multivariate regression analysis for adjustment. All baseline matching and group balancing analyses were performed using SPSS 26.0 statistical software with the built-in case-control matching module. The matching algorithm adopted 1:1 simple nearest-neighbor matching without replacement, with exact matching for sex, an age matching tolerance of ±2 years, and a BMI matching tolerance of ±2 kg/m².

This study adopted simple nearest-neighbor individual matching rather than propensity score matching. No propensity score was estimated, and no propensity score model was established. Baseline balance between the two groups was primarily achieved through the above-mentioned matching criteria (age, sex, BMI), and the balance of baseline characteristics was verified by comparing the demographic and clinical data of the two groups (see **Table 1**), with no significant differences observed in the matching variables between the groups (all P > 0.05), indicating effective matching.

**Table 1:** Comparisons of demographic and laboratory characteristics of the study population.

Variables	Hyperlipidemia group(n=165)	Non-Hyperlipidemia group(n=165)	95% CI	P value
Demographic variables				
Age (years)	44.06 ± 9.69	43.01 ± 9.78	-3.157~1.060	0.329
Sex			0.505~1.208	0.267
Male	98	88		
Female	67	77		
BMI(kg/m2)	25.95 ± 2.55	25.66 ± 3.16	0.377~0.396	0.388
<25	61	73		
≥25	104	92		
Type of symptom			0.828~1.982	0.266
Lumbago	99	89		
Lumbago and Leg pain	66	76		
Symptom duration(month)	25.08 ± 4.01	25.56 ± 3.25	0.162~0.177	0.161
Comorbidities				
Hypertension	24	19	0.686~2.492	0.414
Diabetes mellitus	25	23	0.598~2.034	0.755
Coronary heart disease	20	16	0.640~2.576	0.480
CCI			0.529~2.639	0.683
< 2	151	153		
≥2	14	12		
Lifestyle habits				
Smoking	33	32	0.604~1.788	0.890
Alcohol drinking	29	25	0.665~2.143	0.552
Occupational category			0.531~0.550	0.512
Physical laborers	82	78		
Sedentary individuals	60	69		
Others	23	18		
Lipid-related parameters				
TC(mmol/L)	5.36 ± 1.01	3.91 ± 0.66	-1.630~-1.261	0.000
TG(mmol/L)	2.33 ± 0.96	1.29 ± 0.14	-1.184~-0.888	0.000
LDL-C(mmol/L)	3.40 ± 0.61	3.14 ± 0.45	-0.376~-0.146	0.000
HDL-C(mmol/L)	1.25 ± 0.17	1.31 ± 0.14	0.035~0.103	0.002
APO-A1(g/L)	1.37 ± 0.16	1.43 ± 0.15	0.032~0.099	0.003
APO-B(g/L)	1.17 ± 0.19	1.09 ± 0.19	-0.121~-0.039	0.000
LP(a)(mg/L)	249.64 ± 72.23	219.39 ± 60.30	-44.664~-15.846	0.000
Types of hyperlipidemias				
Hypercholesterolemia(HC)	53	–		–
Hypertriglyceridemia(HTG)	62	–		–
Mixed hyperlipidemia (MHL)	40	–		–
Hypoalphalipoproteinemia (HALP)	10	–		–

### General characteristics and data collection

Baseline data were collected, encompassing age, body mass index (BMI), symptom profile and duration, smoking and alcohol history, and occupational classification (stratified as heavy manual labor: ≥5 hours/day; sedentary office work: 5 hours/day; or other). Comorbidities, including hypertension (HT), diabetes mellitus (DM), and coronary artery disease (CAD), were recorded. The burden of comorbidity was assessed using the Charlson Comorbidity Index (CCI), with higher scores indicating a more severe disease burden.

BMI was calculated as weight (kg) divided by height squared (m²). Based on World Health Organization (WHO) criteria, participants were categorized as overweight (BMI ≥ 25 kg/m²) or obese (BMI ≥ 30 kg/m²) (Akram et al., 2000). Hypertension was defined as a systolic blood pressure ≥ 140 mmHg and/or a diastolic blood pressure ≥ 90 mmHg (Rickenbacher, 2015). The diagnosis of DM was established based on the following criteria: (1) fasting plasma glucose ≥ 7.0 mmol/L; (2) random plasma glucose ≥ 11.1 mmol/L in the presence of typical hyperglycemic symptoms; or (3) glycated hemoglobin (HbA1c) ≥ 6.5% (The American Diabetes Association (ADA), 2020).

The Charlson Comorbidity Index (CCI) was calculated to assess comorbidity burden, with higher scores denoting greater comorbidity severity (Charlson et al., 1987).

### Assessment of hyperlipidemia

The diagnostic criteria for hyperlipidemia were consistent with those described above. The condition was classified into four subtypes:

· Hypercholesterolemia:Elevated serum total cholesterol (TC) with normal triglycerides (TG).

· Hypertriglyceridemia:Elevated serum triglycerides (TG) with normal total cholesterol (TC).

· Mixed hyperlipidemia:Both serum total cholesterol (TC) and triglycerides (TG) are elevated.

· Low high-density lipoprotein cholesterolemia:Reduced serum high-density lipoprotein cholesterol (HDL-C) level (Cleeman et al., 2001).

### Assessment of intervertebral disc degeneration

**Figure 2:**
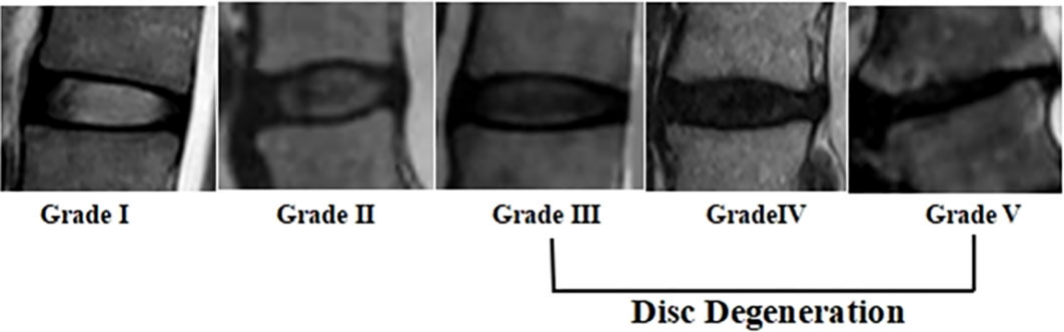
The Pfirrmann score system for assessing lumbar disc degeneration.

The Pfirrmann grading system was employed to evaluate the severity of disc degeneration based on nucleus pulposus signal intensity and disc height, classifying it into five grades: Grade 1: Homogeneous hyperintense (bright white) signal. Grade 2: Inhomogeneous signal with or without a horizontal gray band. Grade 3: Inhomogeneous intermediate (gray) signal. Grade 4: Hypointense (dark gray) signal with near-collapse of the disc. Grade 5: Hypointense signal with complete disc. collapse (**Figure 2**) (Pfirrmann et al., 2001).

Grades 3, 4, and 5 were defined as intervertebral disc degeneration (DD), while grades 4 and 5 were classified as severe degeneration.

Discs from L1/2 to L5/S1 were assessed on mid-sagittal T2-weighted MRI images of the lumbar spine. Multi-level lumbar disc degeneration (MLDD) was defined as degeneration affecting ≥3 disc levels, regardless of whether they were contiguous or non-contiguous (Chang et al., 2022).

Lumbar disc degeneration was evaluated using the Pfirrmann grading system. All MRI images were independently graded by two senior spine surgeons in a double-blinded fashion. The evaluators were blinded to the patients’ clinical data and laboratory results. Inter-observer and intra-observer agreement was assessed using the weighted Kappa statistic, yielding a Kappa value of 0.81 for inter-observer agreement and 0.87 for intra-observer agreement, indicating substantial agreement. Any discrepancies in grading were resolved through discussion to reach a consensus.

### MRI evaluation equipment

A 1.5 T MRI system (Siemens MAGNETOM Symphony) was used for lumbar MRI between L1 and S1. Sagittal T2-weighted images were obtained with the following parameters: TR/TE 3500/88, matrix size 384 × 288, recovery time: 3000–3600 ms, echo time: 80–105 ms, slice thickness: 3.0 mm.

Depending on the specific analytical objectives, this study employed two distinct statistical units:

Patient-level unit: This was used to analyze patient-level characteristics, such as the number of affected discs and the distribution of single-, double-, or multi-level degeneration (**Table 2**).

**Table 2:** The patterns of DD in the study population.

Pattern of DD	Number of Hyperlipidemia patients	Number of non-hyperlipidemia patients	OR	95% CI	P value
Single level	16	39	0.347	0.185~0.650	0.001
Double-level	29	51	0.477	0.284~0.801	0.005
Triple-level	48	58	0.757	0.476~1.203	0.238
Four-level	54	15	4.865	2.611~9.066	0.000
Five-level	18	2	9.980	2.277~43.743	0.000
MLDD	120	75	3.200	2.021~5.067	0.000

Disc-level unit: This was used to analyze disc-level characteristics, including the prevalence of disc degeneration at each segment, the distribution of severe degeneration, and the association between blood lipid indicators and disc degeneration (**Tables 3**–**6**).

**Table 3:** The segmental distribution of lumbar degenerated disc in the study population.

Lumbar disc level	Hyperlipidemia group number of degenerated discs	Non-Hyperlipidemia group number of degenerated discs	OR	95% CI	P value
L1-2	44	9	6.303	2.962~13.415	0.000
L2-3	89	30	5.270	3.196~8.690	0.000
L3-4	132	81	4.148	2.454~6.520	0.000
L4-5	140	128	1.619	0.924~2.837	0.091
L5-S1	147	137	1.669	0.699~3.994	0.112
Total	552	385	2.311	1.893~2.821	0.000

**Table 4:** The segmental distribution of severe lumbar degenerated discs in the study population.

Lumbar disc level	Hyperlipidemia group number of degenerated discs	Non-Hyperlipidemia group number of degenerated discs	OR	95 CI	P value
L1-2	26	2	5.056	0.940~27.194	0.044
L2-3	38	6	2.980	1.109~8.007	0.026
L3-4	47	21	1.580	0.857~2.912	0.141
L4-5	68	52	1.380	0.851~2.240	0.191
L5-S1	71	60	1.199	0.751~1.913	0.447
Total	250	141	1.433	1.097~1.870	0.008

**Table 5:** Association between blood lipid profiles and the count of lumbar degenerated intervertebral disc segments.

Blood lipid test indicators	Indicator status	Number of LDD	Number of non-LDD	OR	95% CI	P value
TC	High	333	157	1.952	1.564~2.438	0.000
	Normal	604	556			
TG	High	357	173	1.921	1.548~2.385	0.000
	Normal	580	540			
HDL-C	Low	31	19	1.250	0.700~2.231	0.519
	Normal	906	694			
LDL-C	High	229	176	0.987	0.787~1.237	0.909
	Normal	708	537			
APO-A1	Low	85	75	0.849	0.612~1.177	0.325
	Normal	852	638			
APO-B	High	266	204	0.989	0.797~1.227	0.921
	Normal	671	509			
LP(a)	High	268	167	1.310	1.047~1.638	0.018
	Normal	669	546			

**Table 6:** Distribution of degenerated lumbar disc segments across four types of hyperlipidemias.

Lumbar disc level	HC	HTG	MHL	HALP	P value
L1-2	11	19	13	1	0.312
L2-3	27	32	25	5	0.666
L3-4	43	49	33	7	0.835
L4-5	48	48	35	9	0.218
L5-S1	47	55	36	9	0.996

### Statistical analysis

All statistical analyses were performed using SPSS 26.0 software. Measurement data were expressed as mean ± standard deviation (x̄ ± s), and the Shapiro-Wilk test was used to test the normality of data distribution. For data that conformed to normal distribution, independent samples t-test was used for comparison between two groups, and one-way analysis of variance (ANOVA) was used for comparison among multiple groups. For data that did not conform to normal distribution, Mann-Whitney U test was used for comparison between two groups, and Kruskal-Wallis H test was used for comparison among multiple groups. Count data were expressed as frequency (n) and percentage (%), and the χ² test was used for comparison between groups.

Multivariate logistic regression analysis was used to adjust for potential confounding factors (including age, sex, BMI, comorbidities, smoking history, drinking history, etc.) to explore the independent association between hyperlipidemia and intervertebral disc degeneration. A P value < 0.05 was considered statistically significant.

## Results

A total of 330 hospitalized patients with low back pain were enrolled in this study to explore the association between hyperlipidemia and lumbar intervertebral disc degeneration. All participants were divided into the case group and the control group, with 165 cases in each group. The demographic, clinical and laboratory characteristics of the two groups are presented in **Table 1**. There were no significant differences between the two groups in terms of age, sex, symptom type, body mass index (BMI), disease duration, occupational distribution, prevalence of hypertension, diabetes mellitus, coronary artery disease, and Charlson Comorbidity Index (CCI) (all P > 0.05).

In the case group, the serum lipid levels were as follows: total cholesterol (TC) 5.36 ± 1.01 mmol/L, triglycerides (TG) 2.33 ± 0.96 mmol/L, high-density lipoprotein cholesterol (HDL-C) 1.25 ± 0.17 mmol/L, low-density lipoprotein cholesterol (LDL-C) 3.40 ± 0.61 mmol/L, apolipoprotein A1 (ApoA1) 1.37 ± 0.16 g/L, apolipoprotein B (ApoB) 1.17 ± 0.19 g/L, and lipoprotein(a) [Lp(a)] 249.64 ± 72.23 mg/L.

In the control group, the corresponding indicators were TC 3.91 ± 0.66 mmol/L, TG 1.29 ± 0.14 mmol/L, HDL-C 1.31 ± 0.14 mmol/L, LDL-C 3.14 ± 0.45 mmol/L, ApoA1 1.43 ± 0.15 g/L, ApoB 1.09 ± 0.19 g/L, and Lp(a) 219.39 ± 60.30mg/L.

Significant differences were observed between the two groups in the levels of TC, TG, HDL-C, LDL-C, ApoA1, ApoB, and Lp(a). (**Table 1**).

To investigate the factors associated with severe lumbar disc degeneration, a binary multivariate logistic regression analysis was conducted, adjusting for age, sex, BMI, symptom characteristics, underlying comorbidities (hypertension, diabetes mellitus, coronary artery disease), lifestyle habits (smoking, alcohol consumption), occupational type, and blood lipid status. This analysis was based on patient-level data, with the dependent variable “severe lumbar disc degeneration” defined as having at least one lumbar disc with Pfirrmann grade 4 or 5, and the independent variable “blood lipids” refers to the state of hyperlipidemia that meets the diagnostic criteria for hyperlipidemia as described in the methods section (dichotomous variable: 1 = hyperlipidemia, 0 = non-hyperlipidemia). To account for the correlated structure of the 1:1 nearest-neighbor non-replacement matched pairs, the multivariable logistic regression model was fitted using cluster-robust standard errors, with each matched pair treated as one cluster to avoid biased standard errors and overly optimistic P values and confidence intervals. As shown in **Table 7**, after adjusting for other confounding factors, hyperlipidemia status was identified as significantly independently associated with severe lumbar disc degeneration (OR = 4.713, 95% CI: 2.645–8.398, P < 0.01). In contrast, no statistically significant associations were observed between severe lumbar disc degeneration and age, sex, BMI, hypertension, diabetes mellitus, coronary artery disease, smoking, alcohol consumption, or occupational type (all P > 0.05). In total, 214 patients were identified as having severe lumbar disc degeneration (at least one disc with Pfirrmann grade 4–5), including 118 in the hyperlipidemia group and 96 in the non-hyperlipidemia group. The total number of outcome events exceeded 120, meeting the conventional statistical rule of 10 events per covariate for multivariate logistic regression. (**Table 7**).

**Table 7:** Binary multivariate logistic regression analysis of factors associated with for severe lumbar disc degeneration.

Variables	Risk of severe lumbar disc degeneration			
	OR	Lower 95% CI	Upper 95% CI	P value
Age	0.986	0.960	1.014	0.325
Sex	0.203	0.041	1.002	0.058
BMI	0.987	0.904	1.076	0.761
Type of symptom	1.266	0.504	3.179	0.615
Symptom duration	1.034	0.961	1.112	0.371
Hypertension	0.998	0.400	2.490	0.996
Diabetes mellitus	1.158	0.538	2.491	0.707
Coronary heart disease	1.526	0.560	4.159	0.409
Smoking	0.994	0.474	2.085	0.988
Alcohol drinking	0.984	0.482	2.008	0.964
Physical laborers				0.206
Sedentary individuals	0.275	0.045	1.676	0.162
Others	0.428	0.162	1.130	0.087
Blood lipids	4.713	2.645	8.398	<0.01

**Table 3** illustrates a significant disparity in the distribution of disc degeneration across lumbar segments between the two cohorts. A significantly greater prevalence of degenerated discs was observed in the hyperlipidemia group within the upper and middle lumbar regions (L1-2, L2-3, and L3-4) than in the non-hyperlipidemia group (all P < 0.001). The number of degenerated discs in the hyperlipidemia group was 44 at L1-2, 89 at L2-3, and 132 at L3-4, compared to 9, 30, and 81, respectively, in the control group.

However, no statistically significant differences were observed in the number of degenerated discs between the two groups at the lower lumbar segments (L4–5 and L5-S1) (P > 0.05). Comprehensive analysis indicated that the total count of degenerated intervertebral discs was notably higher in the hyperlipidemia group (n = 552) than in the non-hyperlipidemia group (n = 385), with a statistically significant difference (P < 0.05). (**Table 3**).

Of note, each patient contained multiple lumbar disc segments, and disc-level observations from the same individual were not statistically independent. All disc-level analyses in **Tables 3**–**6** should be interpreted as exploratory comparative analyses rather than definitive conclusive findings.

In the upper lumbar segments (L1–2 and L2-3), the number of severely degenerated discs was significantly higher in the hyperlipidemia group compared to the non-hyperlipidemia group. Specifically, at the L1–2 level, there were 26 cases in the hyperlipidemia group versus 2 in the non-hyperlipidemia group (P = 0.044); at the L2–3 level, the counts were 38 and 6, respectively (P = 0.026). These differences were statistically significant (P<0.05).

However, no statistically significant differences were observed in the number of severely degenerated discs between the two groups in the middle (L3-4) and lower (L4-5, L5-S1) lumbar segments. The counts at these levels were 47 vs 21 (P = 0.141), 68 vs 52 (P = 0.191), and 71 vs 60 (P = 0.447), respectively, with all P > 0.05.

Comprehensive analysis indicated that the hyperlipidemia group had a significantly greater total number of severely degenerated discs (250) than the non-hyperlipidemia group (141), and this difference was statistically significant (P = 0.008). (**Table 4**).

**Table 2** shows the distribution of disc degeneration patterns in the two groups. In the hyperlipidemia group, 16 patients had single-level disc degeneration, 29 had two-level disc degeneration, 48 had three-level disc degeneration, 54 had four-level disc degeneration, and 18 had five-level disc degeneration, with a total of 120 patients having multi-level disc degeneration. In the control group, 39 patients had single-level disc degeneration, 51 had two-level disc degeneration, 58 had three-level disc degeneration, 15 had four-level disc degeneration, and 2 had five-level disc degeneration, with a total of 75 patients having multi-level disc degeneration. The prevalence of multi-level disc degeneration was significantly higher in the hyperlipidemia group compared to the non-hyperlipidemia group (P = 0.000). (**Table 2**).

**Table 5** presents the correlation analysis between lipid profiles and disc degeneration. In the high TC group, 333 discs exhibited degeneration compared to 157 without significant degeneration. Conversely, in the normal TC group, 604 discs were degenerated and 556 were not. The prevalence of disc degeneration was significantly higher in the high TC group than in the normal group (P = 0.000).

Similarly, regarding TG levels, 357 degenerated discs were observed in the high TG group versus 173 non-degenerated discs, whereas the normal TG group showed 580 degenerated and 540 non-degenerated discs. Disc degeneration was significantly more prevalent in patients with high TG levels (P = 0.000).

For Lp(a), the high Lp(a) group comprised 268 degenerated discs and 167 non-degenerated discs, while the normal Lp(a) group had 669 and 546, respectively. The results indicated a significantly higher prevalence of disc degeneration in the high Lp(a) group compared to the normal group (P = 0.018). (**Table 5**).

**Table 6** shows the segmental distribution of degenerated discs among the four types of hyperlipidemias. There were no significant differences in the distribution of disc degeneration at various lumbar levels among patients with HC, HTG, MHL, or HALP (P = 0.312, P = 0.666, P = 0.835, P = 0.218, P = 0.996). (**Table 6**).

## Discussion

LDD and hyperlipidemia are both highly prevalent clinical conditions that impose significant physical, psychological, and economic burdens on patients. This study enrolled 330 hospitalized patients with low back pain to investigate the association between hyperlipidemia and lumbar disc degeneration. The results demonstrated that patients with hyperlipidemia had a significantly higher prevalence of disc degeneration at the L1/2, L2/3, and L3/4 levels compared to controls. Furthermore, the prevalence of severe degeneration was notably higher in the hyperlipidemia group, particularly at the L1/2 and L2/3 segments. It is worth noting that since the observation results of multiple intervertebral discs in the same patient are not independent of each other, these intervertebral disc-level group differences should be interpreted with caution and regarded as preliminary findings.

Chronic low back pain represents a major medical and social challenge and is a leading cause of disability. Data from the National Center for Health Statistics indicates that approximately 14% of new outpatient visits—amounting to roughly 13 million individuals—are attributed to low back pain (Krock et al., 2014). Disc degeneration is a primary etiology of low back pain, with pain generation mechanisms involving: (1) Disc herniation secondary to degeneration causing mechanical compression of the dorsal root ganglion or spinal nerve roots, resulting in radiculopathy; (2) Upregulation of pro-inflammatory mediators (e.g., IL-1β, TNF-α) and nociceptive mediators (e.g., NGF) within the degenerated disc, which stimulate adjacent nerve fibers; and (3) Disc height reduction and spinal instability leading to inflammation and mechanical wear of adjacent structures, including facet joints, ligaments, and paraspinal muscles (Freemont et al., 2002; Richardson et al., 2012; James et al., 2018).

In healthy adults, the intervertebral disc is avascular; nutrition relies primarily on blood supply from the vertebral body, with only the outer annulus fibrosus receiving vascular supply from surrounding soft tissues (Urban et al., 2004). Consequently, capillary perfusion of the vertebral body is critical for disc health. Nutrients must diffuse from the vertebral body through the cartilaginous endplate into the disc (Grunhagen et al., 2011). Key substances, such as glucose and oxygen, diffuse along cellular metabolic gradients, rendering nucleus pulposus cells highly susceptible to injury due to this precarious nutritional supply system (Sakai and Grad, 2015). Lipid metabolism disorders can impede nutrient transport across the cartilaginous endplate, impair nucleus pulposus cell function, and thereby promote intervertebral disc degeneration (Chang et al., 2022; Chang et al., 2023).

Data from the Committee on the Prevention and Treatment of Dyslipidemia in Chinese Adults indicates that over the past three decades, serum lipid levels in the Chinese population have gradually increased, accompanied by a significant rise in the prevalence of dyslipidemia. A 2012 national survey revealed that the prevalence of hypercholesterolemia, hypertriglyceridemia, and low high-density lipoprotein (HDL) cholesterolemia among adults was 4.9%, 13.1%, and 33.9%, respectively. Consequently, the overall prevalence of dyslipidemia in adults reached 40.4%, and the prevalence of dyslipidemia and related diseases is projected to continue rising (Zhu et al., 2016).

As a systemic disorder, hyperlipidemia can induce atherosclerosis in the lumbar arteries, thereby reducing spinal blood supply, impairing intervertebral disc tissue repair, and accelerating degeneration. Studies on lipid metabolism genes have confirmed that atherosclerosis exacerbates disc degeneration. In an atherosclerotic rabbit model constructed via APOE gene knockout, the number of viable cells in the nucleus pulposus tissue was reduced by 42% compared to normal rabbits. The underlying mechanism may involve atherosclerosis of the abdominal aorta and lumbar arteries impeding nutrient transport, which leads to disc degeneration (Beierfuß et al., 2017).

Furthermore, dyslipidemia may accelerate degeneration through pro-inflammatory factors (Kauppila, 2009; Huang et al., 2022). In a mouse model of disc degeneration induced by a high-fat diet, NF-κB-p65 expression was significantly upregulated. Overactivation of this pathway was shown to reduce type II collagen expression in the disc and regulate the expression of various pro-inflammatory molecules, including TNF-α, IL-1β, IL-6, and IL-8. These findings confirm that hyperlipidemia is a risk factor for intervertebral disc degeneration (Wuertz et al., 2012; Zhang Y. et al., 2019).

The results of this study showed that the number and severity of intervertebral disc degeneration in the hyperlipidemia group were significantly higher than those in the control group. Such intergroup differences were mainly concentrated in the upper lumbar segments including L1/2, L2/3 and L3/4, while no significant differences were observed at the L4/5 and L5/S1 segments.

One possible explanation is that this phenomenon arises from the interaction between biomechanical and metabolic factors. The L4/5 and L5/S1 segments possess the greatest lumbar mobility and bear the highest axial and shear loads, and their intervertebral disc degeneration is predominantly driven by long-term mechanical stress. Accordingly, the prevalence of degeneration in these segments is inherently high in the general population. Against this backdrop, the metabolic impacts induced by hyperlipidemia are masked by the high baseline degeneration rate associated with mechanical loading, rendering additional statistical differences undetectable.

In contrast, the upper lumbar segments (L1/2–L3/4) endure relatively lower mechanical loads, and their degenerative changes are more susceptible to regulation by metabolic factors. Therefore, the promoting effect of metabolic disorders caused by hyperlipidemia on intervertebral disc degeneration is more pronounced in the upper lumbar segments, which contributes to the differential distribution characteristics of degeneration between upper and lower lumbar segments observed in this study. However, this interpretation remains speculative and requires confirmation in future biomechanical and prospective studies.

Lipoprotein(a) [Lp(a)], primarily synthesized in the liver, is a unique cholesterol-rich macromolecular lipoprotein that can infiltrate and deposit within the vessel wall, thereby promoting atherosclerosis. Triglycerides (TG) also constitute a risk factor for atherosclerosis. Both Lp(a) and TG may facilitate disc degeneration through mechanisms such as reducing nutrient supply to the intervertebral disc.

Conversely, elevated total cholesterol (TC) may primarily mediate intervertebral disc cell apoptosis and matrix degradation via the ERK pathway (Zhang X. et al., 2019). High-density lipoprotein (HDL) acts as a protective factor; its established cardiovascular protective effects suggest a potential protective role for the disc, implying that elevating HDL levels might help alleviate lumbar disc degeneration.

Epidemiological evidence supports these associations. A Finnish study identified elevated TC and TG as predictors of low back pain (Leino-Arjas et al., 2006). A large-scale cross-sectional survey of adults aged 40–64 in Japan revealed an association between discogenic low back pain and both low HDL-C levels and a high LDL-C/HDL-C ratio (Yoshimoto et al., 2018). Furthermore, a Chinese case-control study demonstrated a correlation between the TC/HDL-C and LDL-C/HDL-C ratios and disc herniation, noting an increased risk of herniation in individuals with elevated serum LDL-C. These findings suggest that serum lipid profiles may serve as predictors for disc degeneration in the Chinese population.

In the present study, the number of degenerated discs was significantly higher in the elevated TC, TG, and Lp(a) groups compared to the control group. However, no significant associations were observed between disc degeneration and abnormalities in HDL-C, LDL-C, ApoA1, or ApoB. Discrepancies in findings across different regions may be attributed to variations in ethnicity, lifestyle, and methodological approaches.

Our results demonstrated that the prevalence of both single-level and multi-level lumbar disc degeneration was significantly higher in the hyperlipidemia group than in the control group, with 72.73% (120/165) of hyperlipidemic patients exhibiting multi-level degeneration. As a systemic disorder, hyperlipidemia can exert extensive adverse effects on the connective tissues of the spinal discs. Pro-inflammatory cytokines triggered by lipid metabolism disorders may affect discs at multiple levels; consequently, this systemic inflammatory environment is a critical factor contributing to the susceptibility of hyperlipidemic patients to multi-level degeneration.

## Limitations

This study has several limitations. First, as a retrospective observational study based solely on lipid profiles of hospitalized patients, it identifies significant differences in imaging characteristics between groups but cannot establish a definitive causal relationship between hyperlipidemia and disc degeneration. Second, potential confounding factors, such as physical activity, bone mineral density, and education level, were not included in the analysis. Third, although multivariable logistic regression was performed to adjust for several measured confounders, residual confounding from unmeasured factors such as physical activity, bone mineral density, socioeconomic status, and education level cannot be excluded. Finally, since multiple lumbar intervertebral disc segments were analyzed for the same patient, the data from different segments are not independent of each other. Due to the lack of advanced statistical correction methods such as cluster analysis to consider the interdependence within such patients, this may have affected the statistical power of the results. Therefore, the analysis at the intervertebral disc level is an exploratory analysis.

## Conclusion

In summary, this study demonstrates a significant association between hyperlipidemia and lumbar disc degeneration. Among patients hospitalized for low back pain, those with hyperlipidemia exhibited a higher number of degenerated discs, a greater propensity for multilevel degeneration, and a significantly higher proportion of severe degeneration. Specifically, elevated levels of total cholesterol (TC), triglycerides (TG), and lipoprotein(a) [Lp(a)] were found to be correlated with lumbar disc degeneration.

However, the precise pathophysiological mechanisms by which hyperlipidemia and various lipid profiles contribute to the onset and progression of lumbar disc degeneration remain to be fully elucidated. Future research involving larger sample sizes, prospective designs, and basic experimental studies is warranted to further validate the potential impact and clinical utility of lipid profiles in the context of lumbar disc degeneration.

## Data Availability

The original contributions presented in the study are included in the article/supplementary material. Further inquiries can be directed to the corresponding author.
